# Giovanni Gazzinelli (★1927 †2020)

**DOI:** 10.1590/0037-8682-0041-2020

**Published:** 2020-02-21

**Authors:** Walderez O. Dutra

**Affiliations:** 1 Universidade Federal de Minas Gerais, Instituto de Ciências Biológicas, Departamento de Morfologia, Belo Horizonte, MG, Brasil.

“Qual é o problema?” or “Qual é a dúvida?” (“What is the problem?” Or “What is the doubt?”). This is how Professor Giovanni Gazzinelli typically started a conversation with his students and colleagues. Apparently sharp, but always with a half-smile in his face, the phrases reflect his awareness, curiosity and willingness to help us move forward. 

Giovanni Gazzinelli was born in Araçuaí, in the Jequitinhonha region of Minas Gerais, on September 06, 1927. He was the second son of Ettore and Narcy Colares Gazzinelli, and was married for 61 years to Edmea Gazzinelli, whom he addressed as “Darling”, and with whom he had 4 children. He died on January 14, 2020, at 92 years of age and after a prolific life and career. 

Giovanni Gazzinelli, as he was called by his friends and students, started his education in Araçuaí, spent 3 years at the Catholic Seminary in Diamantina, and finished his high school degree in Belo Horizonte. The time at the Seminary, despite short, had a profound impact in some of his most prominent characteristics: discipline and method. Likely influenced by his father, who was a medical doctor, Professor Giovanni enrolled in medical school and graduated as an MD in 1955. He initiated his research in Biochemistry in 1954 under the supervision of Professor José Baeta Vianna, one of the precursors of biochemistry and medical chemistry in Brazil. During the period of 1954-1959 that he worked in the Baeta Vianna’s Lab, he had several lab mates who later became Professors of the Biochemistry and Immunology as well as Physiology Departments at the Federal University of Minas Gerais (UFMG), including Enio Cardillo Vieira, Carlos Diniz, Marcos Luiz dos Mares Guia, Marcus Vinícius Gomez, Fernando Alzamora. During that period, the student Giovanni developed his aptitude for research, and combined his undergraduate studies and laboratory activities with work as a tachygrapher to support his family.

**Figure:**
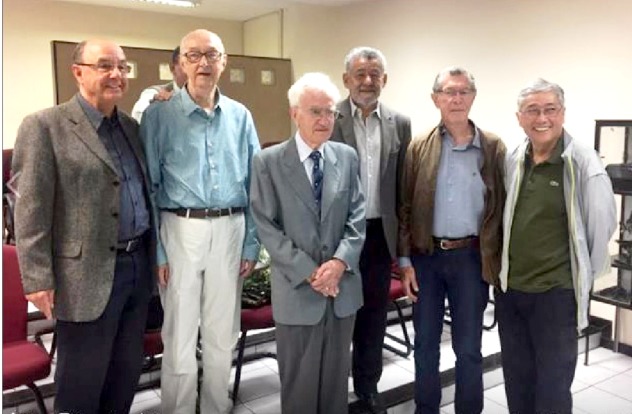
Celebrating his 90th birthday at the Institute of Biological Sciences, UFMG. Left to right: Carlos Alberto Pereira Tavares, Giovanni Gazzinelli, Enio Cardillo Vieira, Tomaz Aroldo Mota Santos, Francisco Juarez Ramalho-Pinto, Elio Hideo Baba.

In 1959, Professor Giovanni received a prestigious Rockefeller fellowship that allowed him to perform a total of three years training in the United States: one year at Tulane University, and two more years in the Department of Biochemistry at the University of Utah, studying protein biosynthesis. During that period, he met several researchers, who later became instrumental collaborators. In 1961, Giovanni published his first paper in the prominent scientific journal *Nature*, entitled “Purification of the toxic fractions from *Ascaris lumbricoides* and their effect on the guinea pig”^1^, as a first author, and Professor Wilmar Dias da Silva was the last author of the work. Professor Wilmar was an instrumental force to set up the biochemistry-immunology partnership at UFMG. Upon his return to Brazil, in December of 1962, Giovanni was hired as an Assistant Professor at the Department of Biochemistry, at the time located at the Medical School of UFMG. In 1965, he obtained his first PhD degree (out of two - the second one obtained in 1972) and, around the same time, the Biochemistry Department was transferred to the newly created Institute of Biological Sciences at UFMG, where Giovanni became a full professor, and where he established his own research group. 

For many years, his research was focused in the study of proteolytic enzymes as well as immunological activity of the complement system, and how these activities guided and influenced parasite-host relationship. His group provided critical contributions to the field of schistosomiasis, particularly in parasite differentiation and scape mechanisms, publishing over 40 papers in the area while at UFMG. He used to say that “studying biochemistry will prepare you to study any other discipline, biochemistry explains it all”. In 1980, Professor Giovanni retired from UFMG and was hired at Instituto René Rachou, FIOCRUZ as a Senior Investigator. In this new phase, he focused his keen intellect and unique insights towards studies in cellular immunology of tropical diseases, mainly human schistosomiasis and Chagas disease. His laboratory was always open and collaborative to Brazilian and foreign researchers. 

Of note was his long-lasting partnership with Dr. Daniel G. Colley, with whom several aspects of immunoregulation and immunopathology of “schisto” and Chagas were unveiled. During this period a large number of students and post-doctoral fellows worked with them and had the opportunity to get additional training in Colley’s lab and other collaborators. Together, they published over 50 papers and developed a close friendship. 

**Figure:**
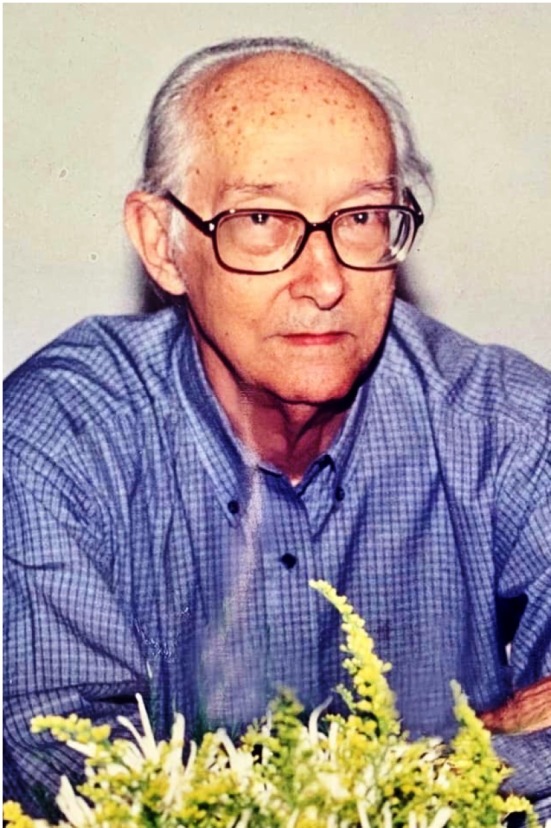

Throughout his outstanding scientific career, Professor Giovanni was granted many prizes and honors. Amongst them, he was a Member of the Brazilian Academy of Sciences and Minas Gerais Academy of Medicine, received the Carlos Chagas and the *Inconfidência* Medals, he was Emeritus Professor at UFMG and Emeritus Researcher at FIOCRUZ, Honorary Member of the American Society of Tropical Medicine and Hygiene, and received the *Grã-Cruz* of the National Order of Scientific Merit. He is internationally recognized for his contributions to the field of immunology of tropical diseases, as exemplified by numerous talks as an invited speaker in international meetings and having held one of the first international collaborative grants funded by the US National Institutes of Health in Brazil. He served as a consultant to the World Health Organization, who also funded many of his projects in the area of human parasitic diseases. Together with colleagues and collaborators such as Aluísio Prata, Zigman Brener, Joaquim Romeu Cançado and João Carlos Pinto Dias he carried out clinical studies in outpatient clinics and endemic areas to study of schistosomiasis and Chagas disease in Minas Gerais. 

Not only Professor Giovanni dedicated himself to science, but he also gave critical contribution to teaching, particularly while at UFMG. He actively participated in the University Reform in 1968, contributed to newly funded medical and biological schools in Barbacena, Pouso Alegre and Itajubá in the State of Minas Gerais, but also outside the state in Teresina (Piauí) and São José do Rio Preto (São Paulo). He firmly believed that the students should guide the learning process with their interest and involvement. As an example, he used to tell us that, in his first biochemistry class, after introducing himself and the discipline, he would announce the chapters that would be discussed in the following theoretical class. In that next class, he would start by asking if the students read the chapters, to which they would typically respond “yes”. He would then ask: “Do you have any questions on the material you read?” if no one presented a question, he would say” “Well, if you don’t have any questions, then the class is over”. He would repeat that same strategy until they started actually asking questions, and by doing so, he would let their interest and involvement guide their classes. 

It is interesting to note that, not only he closely influenced the students he advised in their scientific careers, but also his family has several scientists: his oldest daughters, Andrea and Flavia are full professors at UFMG Nursing School; his younger brothers, Ramayana and Paulo, a retired full professor at UFMG Physics Department and the first Scientific Director of the State of Minas Gerais Research Foundation-FAPEMIG, respectively; his nephews, Ricardo, a full professor at UFMG Biochemistry-Immunology Department and Pedro a post-doctoral fellow at the Immunoparasitology lab at the NIH, as well as his son-in-law, Rodrigo Correa-Oliveira, Vice-President of Research and Biological Collections at FIOCRUZ. 

It is fair to say that Professor Giovanni was one of the pillars of immunology in Brazil. He was one of the founder associates of the Brazilian Society of Immunology. His research and the many students he advised, as well as the next generations, form a significant critical mass of immunologists and immunoparasitologists in Brazil. His assertive style, his meticulousness of scientific method, and his pragmatism - that he also used for several jokes during conversations - certainly marked and strongly impacted his students and collaborators. He was not only a rigorous advisor during the time the students were in his laboratory, but he was also supportive and helpful to them once they continued their carriers: a true mentor. 

 Giovanni’s legacy will be carried on by those whom he advised, and by those who had the privilege of knowing, working and collaborating with him. As the humble person he was, he used to say that the success (not in his words) of his trajectory was “permeated by favorable circumstances” and that he “was lucky”. I dare to say that WE were the lucky ones… 

*This text was written using information obtained from family members, from the FIOCRUZ home page, from former colleagues. I particularly thank prof Enio Cardillo Vieira (who gave the opening talk at GG’s 90th birthday celebration, from where I obtained some information), Rodrigo Correa-Oliveira, Andrea Gazzinelli, and Ricardo Gazzinelli for their input and discussions.*

## References

[1] Gazzinelli G, Mares-Guia M, Neves AGA, Pudles J, Beraldo W, Dias da Silva W (1961). Purification of the toxic fractions from Ascaris lumbricoides and
their effect on the guinea pig.. Nature.

